# Cargo-Mediated Activation of Cytoplasmic Dynein *in vivo*

**DOI:** 10.3389/fcell.2020.598952

**Published:** 2020-10-23

**Authors:** Xin Xiang, Rongde Qiu

**Affiliations:** Department of Biochemistry and Molecular Biology, The Uniformed Services University of the Health Sciences - F. Edward Hébert School of Medicine, Bethesda, MD, United States

**Keywords:** dynactin, cargo adapter, LIS1, early endosome, microtubule plus end, fungi, dynein

## Abstract

Cytoplasmic dynein-1 is a minus-end-directed microtubule motor that transports a variety of cargoes including early endosomes, late endosomes and other organelles. In many cell types, dynein accumulates at the microtubule plus end, where it interacts with its cargo to be moved toward the minus end. Dynein binds to its various cargoes via the dynactin complex and specific cargo adapters. Dynactin and some of the coiled-coil-domain-containing cargo adapters not only link dynein to cargo but also activate dynein motility, which implies that dynein is activated by its cellular cargo. Structural studies indicate that a dynein dimer switches between the autoinhibited phi state and an open state; and the binding of dynactin and a cargo adapter to the dynein tails causes the dynein motor domains to have a parallel configuration, allowing dynein to walk processively along a microtubule. Recently, the dynein regulator LIS1 has been shown to be required for dynein activation *in vivo*, and its mechanism of action involves preventing dynein from switching back to the autoinhibited state. In this review, we will discuss our current understanding of dynein activation and point out the gaps of knowledge on the spatial regulation of dynein in live cells. In addition, we will emphasize the importance of studying a complete set of dynein regulators for a better understanding of dynein regulation *in vivo*.

## Introduction

In eukaryotic cells, motor proteins such as dyneins, kinesins and myosins are ATPases, and they use the energy from ATP hydrolysis to drive intracellular motility (Hirokawa et al., 2009; Verhey and Hammond, 2009; Dodding and Way, 2011; Hammer and Sellers, 2011; Reck-Peterson et al., 2018; Olenick and Holzbaur, 2019; Scherer et al., 2020). In most mammalian cells, polarized microtubules serve as tracks for long-distance transport: while the plus-end-directed kinesins transport cargoes toward the microtubule plus ends near the cell periphery, the minus end-directed cytoplasmic dynein transports cargoes inward from the cell periphery (Reck-Peterson et al., 2018). Cytoplasmic dynein-1 (called “dynein” hereafter for simplicity) powers the intracellular transport of nuclei/mitotic spindles, Golgi, mitochondria, early endosomes, late endosomes, autophagosomes, proteins, mRNAs and/or virus particles (Dodding and Way, 2011; Reck-Peterson et al., 2018; Olenick and Holzbaur, 2019; Scherer et al., 2020). Deficiencies in dynein and its regulators such as dynactin and LIS1 (Lissencephaly-1) cause devastating neurodegenerative diseases and brain developmental disorders (Wynshaw-Boris, 2007; Maday et al., 2014; Guedes-Dias and Holzbaur, 2019; Markus et al., 2020).

Studies have shown that dynein binds to its various cargoes via the dynactin complex and cargo adapters. Importantly, some cargo adapters not only link dynein to cargos but also activate dynein motility, which implies that the dynein motor is activated by its cargo (Reck-Peterson et al., 2018; Olenick and Holzbaur, 2019; Canty and Yildiz, 2020). While some kinesin and myosin motors are also known to be activated by cargo or specific adapters/scaffolding proteins (Sellers and Knight, 2007; Trybus, 2008; Hirokawa et al., 2009; Verhey and Hammond, 2009; Fu and Holzbaur, 2014; Sweeney and Holzbaur, 2018), the mechanism of dynein activation is distinct due to the unique structure of the dynein motor. In this review, we will cover our current understanding on the mechanism of dynein activation. We will mainly use early endosome transport in filamentous fungi as an example to discuss how dynein activation is spatially regulated *in vivo* and point out unresolved issues that need to be addressed in the future.

## Dynactin and Cargo Adapter Proteins Mediate the Dynein-Cargo Interaction

**FIGURE 1:**
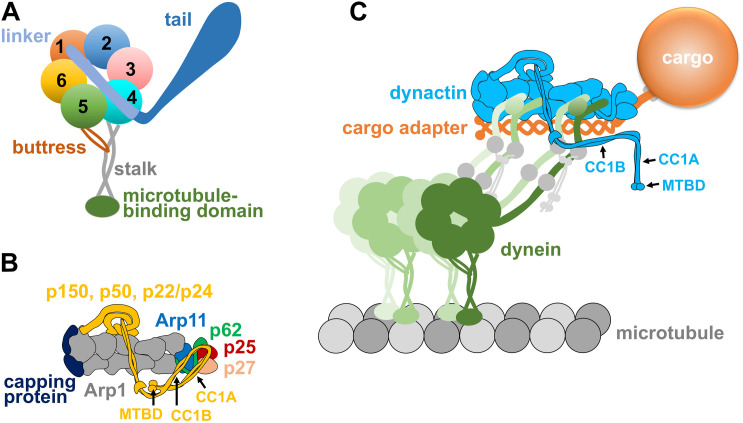
**(A)** A diagram of the dynein heavy chain with a motor ring containing six AAA domains (1–6), a linker domain connected to the beginning of AAA1 and the N-terminal tail domain connected to the linker. A microtubule-binding domain is connected to the coiled-coil stalk that emerges from a location between AAA4 and AAA5, and the buttress coming from AAA5 supports the stalk. All these domains work together to ensure minus-end-directed motility of dynein as well as other properties of the dynein motor such as tension sensing (Roberts et al., 2013; Carter et al., 2016; Can et al., 2019; Rao et al., 2019). **(B)** A diagram of the dynactin complex with its Arp1 mini-filament, barbed-end capping protein, pointed-end proteins Arp11, p62, p25, and p27, as well as the shoulder/sidearm proteins p150, p22/p24, and p50 (Schroer, 2004; Urnavicius et al., 2015). Note that p150 is depicted as a folded molecule, and its CC1A, CC1B, and microtubule-binding domain (MTBD) are indicated by arrows. **(C)** A diagram of the dynein-dynactin-cargo adapter complex and the cargo linked to the cargo adapter. The cargo adapter, depicted as a homodimer, contains coiled-coil domains. Note that the dynein tails bind to the Arp1 mini-filament of dynactin, as shown by EM structural analyses (Schlager et al., 2014; Chowdhury et al., 2015; Urnavicius et al., 2015). An extended form of p150 is depicted, and its CC1A, CC1B, and microtubule-binding domain (MTBD) are indicated by arrows. Two dynein dimers are depicted, since a fraction of the dynein-dynactin-cargo adapter complexes could contain a second dynein dimer along the Arp1 mini-filament (Grotjahn et al., 2018; Urnavicius et al., 2018). Dynein intermediate chains and dynein light intermediate chains are depicted as gray circles on the dynein tails, and dynein light chains are depicted as small gray circles connected to the dynein intermediate chains.

Compared to kinesins or myosins, dynein is extremely huge and complex (Höök and Vallee, 2006; Sweeney and Holzbaur, 2018). It is a multi-protein complex of ∼1.4 MDa containing two dynein heavy chains (HCs) as well as other subunits such as intermediate chains (ICs), light intermediate chains (LICs), and light chains (LCs) (Pfister et al., 2005; Reck-Peterson et al., 2018). The HCs form a homodimer, and each HC monomer contains the C-terminal motor head and the N-terminal tail. The motor head is responsible for motility, while the tail is responsible for HC-HC dimerization and also binds other dynein subunits as well as the dynactin complex (King, 2000; Carter et al., 2016; Schmidt and Carter, 2016). The motor head of the dynein HC consists a motor ring with six AAA (ATPases Associated with diverse cellular Activities) domains, a linker (∼10 nm) connecting the motor ring with the tail (Burgess et al., 2003), and a microtubule-binding domain that is connected to the motor ring via a coiled-coil stalk extending out between AAA4 and AAA5 (Gee et al., 1997; Gibbons et al., 2005; Cianfrocco et al., 2015; Figure 1A). ATP binding and hydrolysis at AAA1 cause conformational changes in the ring, which can be transmitted via the coiled-coil stalk to the microtubule binding site, driving dynein movement along a microtubule (Roberts et al., 2013; Carter et al., 2016). ATP hydrolysis at AAA3 allows the proper transmission of conformational changes around the ring, allowing dynein to be released from the microtubule when ATP is bound to AAA1 (Bhabha et al., 2014; Dewitt et al., 2015; Nicholas et al., 2015).

The dynactin complex of ∼1 MDa is involved in almost all functions of cytoplasmic dynein (Schroer, 2004). The backbone of the dynactin complex is an Arp1 mini-filament of ∼37 nm (Schafer et al., 1994), which provides the binding sites for dynein tails and cargo adapters (Chowdhury et al., 2015; Urnavicius et al., 2015). The pointed end of the Arp1 mini-filament is occupied by the pointed-end subcomplex containing p25, p27, p62 and Arp11 (Eckley et al., 1999; Figure 1B). The barbed end of the Arp1 mini-filament is occupied by the actin-capping protein, which also caps the barbed ends of conventional actin filaments (Schafer et al., 1994; Wear and Cooper, 2004). The largest subunit of the dynactin complex is p150^*Glued*^ (or p150 for simplicity), which contains a microtubule-binding domain (MTBD) at its N-terminus (Holzbaur et al., 1991; Waterman-Storer et al., 1995). Following the MT-binding domain are the coiled-coil domains CC1 and CC2, and CC1 interacts with the N-terminus of dynein IC in biochemical assays (Karki and Holzbaur, 1995; Vaughan and Vallee, 1995; King et al., 2003). The CC1 domain of p150 can be further divided into CC1A and CC1B (Figures 1B,C), and CC1B contains a dynein-IC-binding domain (McKenney et al., 2011; Loening et al., 2020) whose function may be modulated by the binding of CC1A (Tripathy et al., 2014; Saito et al., 2020). Two other subunit of the dynactin complex, p22/p24 and p50 dynamitin that forms an oligomer (Echeverri et al., 1996; Karki et al., 1998; Melkonian et al., 2007), together with part of the p150 subunit, form a “shoulder/side arm” adjacent to the Arp1 mini-filament, and the p50 oligomer was proposed to function as a template for Arp1 mini-filament assembly (Urnavicius et al., 2015). Interestingly, in the dynein-dynactin cargo adapter tripartite complex, it is the Arp1 mini-filament rather than the p150 subunit that interacts with the tails of dynein (Schlager et al., 2014; Chowdhury et al., 2015; Urnavicius et al., 2015, 2018; Grotjahn et al., 2018; Figure 1C). Future work will be needed to address whether the dynein IC-p150 interaction found by biochemical assays is important for initiating the dynein-dynactin interaction before cargo adapter binding.

The dynactin complex is needed for the dynein-cargo interaction. This function of dynactin was proposed many years ago (Schroer and Sheetz, 1991; Waterman-Storer et al., 1997), but this idea had remained controversial (Haghnia et al., 2007). This subject was revisited in two independent studies on the pointed-end proteins of the Arp1-minifilament, in the filamentous fungus *Aspergillus nidulans* and mammalian cells (Zhang et al., 2011; Yeh et al., 2012). These studies have found that the pointed-end proteins p25 in *A. nidulans* and the p25/p27 heterodimer in mammalian cells play a critical role in the interaction of dynein with its early endosome cargo (Zhang et al., 2011; Yeh et al., 2012). Both p25 and p27 adopt a left-handed beta-helix structure (Parisi et al., 2004; Yeh et al., 2013). Because p25 has many hydrophobic residues, it seemed plausible that p25 may contact membrane directly (Yeh et al., 2012). However, follow-up genetic screens in *A. nidulans* as well as another filamentous fungus *Ustilago maydis* led to the discovery that the FTS-Hook-FHIP (FHF) complex (Xu et al., 2008) functions as an adapter allowing dynein-dynactin to link with early endosomes (Bielska et al., 2014b; Yao et al., 2014; Zhang et al., 2014).

The Hook proteins (three in mammalian cells: Hook1, Hook2 and Hook3) and the FHF complex were initially discovered in higher eukaryotic cells (Krämer and Phistry, 1996; Walenta et al., 2001; Xu et al., 2008). Within the fungal FHF complex, HookA in *A. nidulans* and Hok1 in *U. maydis* use their N-terminal Hook domain (Schroeder and Vale, 2016) and the coiled-coil domains to interact with dynein-dynactin (Bielska et al., 2014b; Zhang et al., 2014; Qiu et al., 2018), and this interaction depends on Arp1 and p25 (Zhang et al., 2014). Structural studies on the mammalian Hook3 protein further demonstrate that a Hook protein binds dynein-dynactin directly via dynein light intermediate chain (Schroeder and Vale, 2016; Lee et al., 2018) and the Arp1 filament (Urnavicius et al., 2018). The interaction of fungal hook proteins with the early endosome depends on FTS and FHIP (Yao et al., 2014; Guo et al., 2016); FHIP makes the closest contact with early endosome (Yao et al., 2014), most likely via its direct interaction with Rab5 (Guo et al., 2016). It is unclear whether FTS and FHIP are involved in targeting mammalian Hook1, Hook2, or Hook3 onto other cargoes or cellular structures, such as the TrkB–BDNF-signaling endosome (Olenick et al., 2019), nuclear envelope (Dwivedi et al., 2019b), centrosome (Szebenyi et al., 2007a), aggresome (Szebenyi et al., 2007b), and the Golgi apparatus (Walenta et al., 2001). It should be pointed out that Hook1 interacts directly with cargo proteins of the recycling endosomes (Maldonado-Báez et al., 2013), and Hook1 and Hook2 bind directly with AP4 (adaptor protein complex 4 of the *trans*-Golgi network), which is responsible for trafficking of the autophagy protein ATG9A (Mattera et al., 2020).

There are several other important dynein adapters (Reck-Peterson et al., 2018; Olenick and Holzbaur, 2019), such as proteins of the Bicaudal D (BICD) family including BicD2 (Hoogenraad and Akhmanova, 2016), Rab11-FIP3 (Horgan et al., 2010) and Spindly (Griffis et al., 2007). The domain organization of these dynein adapter proteins are similar in that they all contain an N-terminal portion including the coiled-coil domains important for binding dynein-dynactin and a C-terminus required for cargo binding (Reck-Peterson et al., 2018; Dwivedi et al., 2019a; Olenick and Holzbaur, 2019). *In vitro* experiments show that the N-terminal portion of the cargo adapters can enhance the interaction between the dynein complex and the dynactin complex (Splinter et al., 2012; McKenney et al., 2014; Olenick et al., 2016). Such an effect was first shown for the N-terminal part of the BicD2 protein (Splinter et al., 2012), a result highly instrumental to the ground-breaking experiments revealing cargo adapters being critical for dynein activation (McKenney et al., 2014; Schlager et al., 2014).

## Dynactin and Specific Cargo Adapter Proteins Activate Dynein

Mammalian dynein by itself is incapable of moving along the microtubule processively, although the dynein is active in a microtubule-gliding assay (Trokter et al., 2012; McKenney et al., 2014; Schlager et al., 2014). Adding dynactin alone does not seem to help even when dynactin is added in large excess (McKenney et al., 2014; Schlager et al., 2014). Astonishingly, addition of both BicD2 and dynactin enhances the processivity of dynein dramatically (McKenney et al., 2014; Schlager et al., 2014). Importantly, not only the BicD2 N-terminus but also the dynein-dynactin-binding portion of Hook3 and other cargo adapters with a similar domain organization, including Rab11-FIP3 that targets dynein to Rab11-positive vesicles (Horgan et al., 2010) and Spindly that targets dynein to kinetochores (Griffis et al., 2007; Gama et al., 2017), all stimulated dynein processivity via an enhancement of the dynein-dynactin interaction (McKenney et al., 2014). The processivity and velocity of dynein motility activated by the N-terminal Hook1 and Hook3 are even higher than that by the N-terminal BicD2 (Olenick et al., 2016). These coiled-coil domain-containing dynein adapters are considered as “activating adapters,” and new dynein activators with this domain signature have continuously been discovered (Redwine et al., 2017; Reck-Peterson et al., 2018; Olenick and Holzbaur, 2019; Wang et al., 2019).

As revealed by a cryo-EM study, dynein activation results from a conformational change of dynein upon binding to dynactin and a cargo adapter (Zhang K. et al., 2017). Specifically, the two dynein heavy chains within the dimer are initially held in an auto-inhibited “phi” conformation with the two heavy chains positioned very close to each other (Amos, 1989; Torisawa et al., 2014; Zhang K. et al., 2017). This conformation can be switched to an “open” state with the two dynein heavy chains separated from each other. Although the “open dynein” has a higher affinity for microtubules, it is still not configured properly to move along a microtubule (Zhang K. et al., 2017). Only after the dynein tails bind dynactin and a cargo adapter, the two motor domains with the microtubule-binding stalks become parallel, which allows processive movement (Zhang K. et al., 2017). It is important to point out that since dynactin and cargo adapters bind dynein tails (Schlager et al., 2014; Chowdhury et al., 2015; Urnavicius et al., 2015; Zhang K. et al., 2017), the conformational change in the dynein motor domains must be transmitted through the dynein tails. This may explain why changes in the dynein tail, especially subtle mutations that do not affect the dynein-dynactin interaction, can lead to a severe defect in dynein function (Ori-McKenney et al., 2010; Sivagurunathan et al., 2012; Qiu et al., 2013; Hoang et al., 2017; Marzo et al., 2019).

EM-based structural studies have shown that in some dynein-dynactin-cargo adapter complexes, there are two dynein dimers instead of one, and both dimers have their tails positioned along the Arp1 mini-filament (Grotjahn et al., 2018; Urnavicius et al., 2018). Some cargo adapters including Hook3 and BicDR1 have a much stronger tendency to form this type of complexes with an extra dynein compared to other cargo adapters such as BicD2, and the presence of two dynein dimers enhances dynein’s speed and force output (Urnavicius et al., 2018). This may be part of the reason why some of the dynein-dynactin-Hook3 complexes move with a higher velocity compared to the dynein-dynactin-BicD2 complexes (Olenick et al., 2016). Recently, the dynein regulator LIS1 has also been shown to enhance the recruitment of the second dynein dimer to the dynein-dynactin-cargo adapter complex *in vitro* (Elshenawy et al., 2020; Htet et al., 2020), further suggesting the importance of the second dynein dimer. However, it still remains to be determined what proportion of cargo-bound dynactin in live cells contain two dynein dimers associated with the Arp1 mini-filament and how different regulators change this proportion.

While the *in vitro* motility studies and structural analysis provided significant insights into the mechanism of dynein activation, knowledges gained from *in vivo* studies further shed light on the spatial regulation of dynein activity. In filamentous fungi including *Aspergillus nidulans* and *Ustilago maydis*, the dynamic microtubule plus ends face the hyphal tip, and both dynein and dynactin are strongly enriched at the microtubule plus ends (Han et al., 2001; Zhang et al., 2003; Lenz et al., 2006). Fungal dynein transports many cargoes and its major cargo is the early endosome, which undergo rapid bi-directional movements (Wedlich-Soldner et al., 2002; Lenz et al., 2006; Abenza et al., 2009; Zekert and Fischer, 2009; Penalva et al., 2017; Hernández-González et al., 2018; Otamendi et al., 2019; Bieger et al., 2020). Early endosome motility not only is coupled to endosome maturation (Abenza et al., 2010, 2012), it also helps distribute hitchhiking cargoes including peroxisomes, ribosomes and RNAs (Baumann et al., 2012; Bielska et al., 2014a; Higuchi et al., 2014; Guimaraes et al., 2015; Pohlmann et al., 2015; Lin et al., 2016; Salogiannis et al., 2016). Early endosomes are moved by kinesin-3 toward the plus ends near the hyphal tip and then delivered to dynein to be moved away from the hyphal tip (Lenz et al., 2006). The accumulation of dynein at the microtubule plus end depends on kinesin-1 and dynactin (especially the microtubule-binding domain of p150) (Xiang et al., 2000; Zhang et al., 2003; Lenz et al., 2006; Egan et al., 2012; Yao et al., 2012), and dynein-mediated early endosome transport depends on kinesin-1, most likely because the accumulation of dynein at the plus end enhances the chance for the dynein-early endosome interaction (Lenz et al., 2006; Zhang et al., 2010).

**FIGURE 2:**
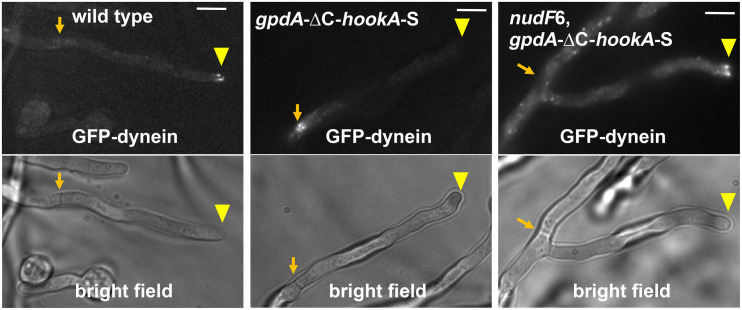
Dynein activation in *A. nidulans* depends on NudF/LIS1. In wild-type cells, dynein is accumulated at the microtubule plus ends, and this accumulation is represented by the comet-like structures formed by GFP-labeled dynein near the hyphal tip (Xiang et al., 2000; Han et al., 2001). Dynein activation, as judged by dynein relocation from the microtubule plus ends at hyphal tip (yellow arrowhead) to the minus ends at septum (Zhang Y. et al., 2017) (brown arrow), is driven by the dynein-dynactin-binding portion of the cargo adapter HookA, ΔC-HookA, overexpressed under the *gpdA* promoter (*gpdA*-ΔC-*hookA*-S, note that “S” indicates S-tag, an affinity tag for biochemical studies) (Qiu et al., 2019). In the *nudF*6 mutant, a NudF/LIS1 loss-of-function mutant, dynein is retained at the microtubule plus ends. Bright-field images are shown below to indicate hyphal shape and septal position. Bars, 5 μm. These images have been published previously in the Journal of Cell Biology (Qiu et al., 2019).

Given the current understanding of dynein’s structural change during its activation (Zhang K. et al., 2017), we speculate that fungal dynein is in the autoinhibited phi conformation while being transported by kinesin-1 toward the microtubule plus end. This would prevent a tug-of-war between kinesin-1 and dynein. Conceptually, this is similar to the regulatory mechanism of dynein-2 in intraflagellar transport (IFT) as revealed by cryo-electron tomography: dynein-2 is in an autoinhibited conformation when it is being transported to the plus end by kinesin-2 (Jordan et al., 2018). In *A. nidulans* and *U. maydis*, the microtubule plus end-localized dynein-dynactin interact with early endosomes on which the activating cargo adapter HookA or Hok1 is bound (Bielska et al., 2014b; Zhang et al., 2014). In *A. nidulans*, overexpression of the cytosolic ΔC-HookA drives dynein departure from the microtubule plus ends and causes dynein to accumulate at the minus ends (Qiu et al., 2019; Figure 2). This supports not only the idea of cargo adapter-mediated dynein activation emerged from *in vitro* studies (McKenney et al., 2014; Schlager et al., 2014) but also the postulation that the plus-end dynein is activated by its early endosome cargo (Lenz et al., 2006).

The activating function of the cargo adapters has been thought to be involved in the enhancement of the dynein-dynactin interaction. Interestingly, the dynein-dynactin interaction must have occurred to a certain extent before cargo binding in cells. In filamentous fungi, dynactin is required for the plus-end localization of dynein (Xiang et al., 2000; Zhang et al., 2003, 2008; Lenz et al., 2006; Egan et al., 2012; Yao et al., 2012), suggesting that either the two complexes are transported together by kinesin-1 to the plus end or they associate at the plus end after being transported separately. It seems likely that they are transported together because loss of Arp1 reduced the interaction between dynein and kinesin-1 (Qiu et al., 2018). HookA is able to interact with dynein or dynactin only when both complexes are present (Zhang et al., 2014), although dynein LIC binds the Hook domain directly (Malone et al., 2003; Schroeder and Vale, 2016; Lee et al., 2018). Thus, it is most likely that an early endosome interacts with a plus end dynein-dynactin complex and changes the configuration of the dynein-dynactin interaction to make it productive for minus-end-directed movement. This notion is consistent with previous findings that while the dynein IC in the dynein tail interacts with the p150 subunit of dynactin without cargo adapter (Karki and Holzbaur, 1995; Vaughan and Vallee, 1995; King et al., 2003), the dynein tails bind the Arp1 filament in the presence of cargo adapters (Schlager et al., 2014; Chowdhury et al., 2015; Urnavicius et al., 2015, 2018; Zhang K. et al., 2017; Grotjahn et al., 2018).

The spatial regulation of dynein activation appears to be evolutionarily conserved. In Drosophila oocyte, and in *Caenorhabditis elegans* and mammalian neurons, kinesin-1 has been implicated in transporting dynein toward the microtubule plus ends for function (Brendza et al., 2002; Duncan and Warrior, 2002; Januschke et al., 2002; Palacios and St Johnston, 2002; Yamada et al., 2008, 2010; Arimoto et al., 2011; Twelvetrees et al., 2016). The kinesin-1-dynein interaction has been dissected in detail, and it was found that the dynein intermediate chain interacts directly with the light chains of kinesin-1 in mammalian hippocampal neurons (Ligon et al., 2004; Twelvetrees et al., 2016). In C. *elegans*, however, the interaction between kinesin-1 and dynein is mediated by UNC-16 that binds to the dynein light intermediate chain (Arimoto et al., 2011). In mouse DRG neurons, dynactin and dynein are transported separately by kinesin-1 via mNudC (Yamada et al., 2010). Thus, how kinesin-1 transports dynein may differ in different cell types. What appears to be conserved is the need to get dynein-dynactin to the microtubule plus end using the plus-end-directed kinesin-1, which could enhance the chance of dynein-cargo interaction. In mammalian and Drosophila neurons, the microtubule-binding domain of p150 dynactin is required for enriching dynactin at the distal end of an axon, thereby facilitating the initiation of retrograde transport from the neurite tip or synaptic termini (Lloyd et al., 2012; Moughamian and Holzbaur, 2012). Hook1, which is able to activate dynein (Olenick et al., 2016), is required for transporting signaling endosomes in axons (Olenick et al., 2019).

In the budding yeast, dynein is almost exclusively used for moving nuclei/spindles (Eshel et al., 1993; Li et al., 1993; Winey and Bloom, 2012), and both dynein and dynactin are clearly accumulated at the microtubule plus end (Lee et al., 2003; Sheeman et al., 2003; Moore et al., 2008). Although dynein can be recruited directly from a cytoplasm to the plus end via Bik1/Clip170 and Pac1/LIS1, Kip2 (kinesin-7) also plays an important role in transporting Bik1/Clip170 and dynein to the microtubule plus end (Lee et al., 2003; Sheeman et al., 2003; Carvalho et al., 2004; Markus et al., 2011; Roberts et al., 2014). Dynein accumulated at the microtubule plus end is activated by its cortical anchor Num1, which is a cargo adapter-like molecule containing coiled-coil domains (Farkasovsky and Küntzel, 1995; Lee et al., 2003; Sheeman et al., 2003; Markus and Lee, 2011; Tang et al., 2012; Lammers and Markus, 2015). In the fission yeast, dynein drives the oscillatory movement of meiotic prophase nucleus (Yamamoto et al., 1999), and this function of dynein depends on dynactin and the Num1-like cortical anchor (Niccoli et al., 2004; Yamashita and Yamamoto, 2006). It was found that dynein molecules along microtubules are inactive but activated by the cortical Num1 homolog (Ananthanarayanan et al., 2013). Thus, although yeast dynein is active on its own *in vitro* (Reck-Peterson et al., 2006), yeast dynein’s cortical anchor functions as an activating cargo adapter *in vivo*.

## Lis1 Is a Positive Regulator for Dynein Activation

Beside dynactin and cargo adapters, another important protein involved in dynein activation is LIS1 (Lissencephaly-1) (Markus et al., 2020). The mechanism of LIS1 action in the dynein pathway has been controversial, but multiple recent studies suggest that LIS1 promotes the open dynein conformation, thereby facilitating dynein activation (Qiu et al., 2019; Elshenawy et al., 2020; Htet et al., 2020; Marzo et al., 2020; McKenney, 2020). LIS1, a WD40-repeats-containing protein, was initially identified as a causal gene for type 1 lissencephaly, a human brain developmental disorder (Reiner et al., 1993). Its functional connection to dynein was first suggested by genetic studies in fungi and further demonstrated in higher eukaryotic organisms and cell types (Xiang et al., 1995; Geiser et al., 1997; Liu et al., 1999, 2000; Faulkner et al., 2000; Lei and Warrior, 2000; Smith et al., 2000; Markus et al., 2020). In contrast to dynactin that binds to the dynein tail (Karki and Holzbaur, 1995; Vaughan and Vallee, 1995; Chowdhury et al., 2015; Urnavicius et al., 2015), LIS1 binds directly to the dynein motor ring at AAA3/AAA4 as shown by cryo-EM studies (Huang et al., 2012; Toropova et al., 2014; Desantis et al., 2017; Htet et al., 2020). LIS1’s binding to this site is not compatible with the autoinhibited phi conformation of dynein (Htet et al., 2020; Marzo et al., 2020), which supports a “check valve” (Markus et al., 2020) model of LIS1 mechanism of action: it stabilizes the open dynein conformation and prevents it from switching to the autoinhibited phi state, thereby facilitating cargo-adapter-mediated dynein activation (Qiu et al., 2019; Canty and Yildiz, 2020; Elshenawy et al., 2020; Htet et al., 2020; Markus et al., 2020; Marzo et al., 2020).

In budding yeast and mammalian cells, LIS1 is required for the microtubule plus-end accumulation of dynein and consequently dynein offloading to cortex or cargoes (Lee et al., 2003; Sheeman et al., 2003; Markus and Lee, 2011; Markus et al., 2011; Splinter et al., 2012; Tame et al., 2014). However, dynactin rather than LIS1 plays a critical role in the plus-end accumulation of dynein in filamentous fungi (Zhang et al., 2003; Lenz et al., 2006; Egan et al., 2012). This allows the role of LIS1 in cargo-adapter-mediated dynein activation *in vivo* to be shown clearly in *A. nidulans* (Qiu et al., 2019). Specifically, while overexpression of ΔC-HookA drives almost a complete relocation of dynein from the microtubule plus ends to the minus ends, dynein remains at the plus ends in the *nudF* (lis1) loss-of-function mutants when ΔC-HookA is overexpressed (Qiu et al., 2019; Figure 2). This requirement of LIS1 for HookA-mediated dynein activation *in vivo* is consistent with a role of LIS1 in enhancing the frequency of departure of the dynein-dynactin-cargo adapter complex from the microtubule plus end *in vitro* (Baumbach et al., 2017; Jie et al., 2017). Importantly, a phi mutation that promotes open dynein (Zhang K. et al., 2017) bypasses the requirement for LIS1 to a significant extent (Qiu et al., 2019), suggesting that LIS1 is involved in promoting open dynein, thereby facilitating cargo adapter-mediated dynein activation (Qiu et al., 2019). This main conclusion agrees with three other recent studies (Elshenawy et al., 2020; Htet et al., 2020; Marzo et al., 2020), although LIS1 in *A. nidulans* does not seem to significantly affect the formation of the dynein-dynactin-ΔC-Hook complex (Qiu et al., 2019), while LIS1 enhances the recruitment of the second dynein to the dynein-dynactin-BicD2N complexes *in vitro* (Elshenawy et al., 2020; Htet et al., 2020). Possibly, *A. nidulans* LIS1 still enhances the dynein-dynactin interaction as described in other systems (Dix et al., 2013; Wang et al., 2013), but this effect was not easily detected when the concentration of cytosolic cargo adapters is high enough. We should also point out that LIS1 may play roles beyond stabilizing the open dynein because constitutively opening dynein does not allow the requirement for LIS1 to be completely bypassed (Qiu et al., 2019; Elshenawy et al., 2020; Htet et al., 2020; Marzo et al., 2020).

**FIGURE 3:**
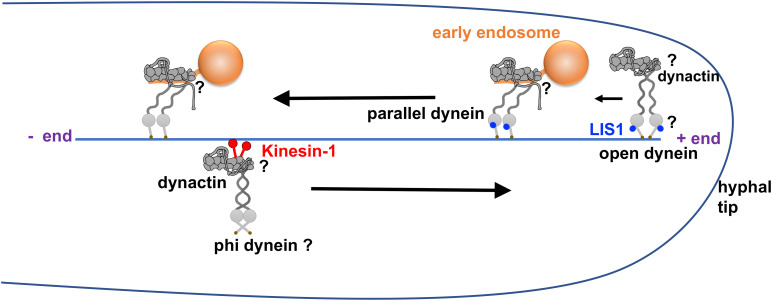
Model of dynein activation in filamentous fungi. This model is based on the structural data on the LIS1-dynein interaction and cargo-adapter-mediated dynein activation (Huang et al., 2012; Zhang K. et al., 2017; Htet et al., 2020), live cell imaging data on dynein, dynactin and LIS1 from filamentous fungi (Han et al., 2001; Zhang et al., 2003; Efimov et al., 2006; Lenz et al., 2006; Egan et al., 2012; Yao et al., 2012) as well as recent data on LIS1 mechanism (Qiu et al., 2019; Elshenawy et al., 2020; Htet et al., 2020; Marzo et al., 2020). In this model, dynein in the autoinhibited phi conformation is transported by kinesin-1 to the microtubule plus end together with dynactin. At the microtubule plus end, the dynein-LIS1 interaction keeps the motor domains of the dynein dimer in an open configuration but still being incapable of processive movement until dynein-dynactin interacts with an early endosome cargo. The cargo adapter (HookA or Hok1) on the early endosome interacts with dynein-dynactin-LIS1 and switches the dynein dimer into a parallel configuration, allowing it to walk toward the microtubule minus end. Note that there is also a conformational change of dynactin after cargo adapter binding so that the p150 protein is in an extended conformation (Urnavicius et al., 2015). Finally, LIS1 dissociates from dynein during the minus-end-directed movement (Lenz et al., 2006; Egan et al., 2012). The questions marks in this figure indicate the speculative nature of the dynein-dynactin configurations at these sites.

In filamentous fungi and budding yeast, LIS1 accumulates at the microtubule plus end just like dynein (Han et al., 2001; Lee et al., 2003; Callejas-Negrete et al., 2015). LIS1’s plus-end accumulation depends partly on dynein, its binding partner NudE as well as the CLIP170 homolog CLIPA or Bik1 (Zhang et al., 2003; Li et al., 2005; Efimov et al., 2006; Markus et al., 2011). This is consistent with earlier data from mammalian cells and budding yeast indicating a direct interaction between LIS1 and CLIP170/Bik1 (Coquelle et al., 2002; Sheeman et al., 2003). In the budding yeast, the dynein-LIS1-Bik1/CLIP170 complex could be transported by the Kip2 kinesin-7 to the microtubule plus end, or, dynein and LIS1 form a complex before being directly recruited from the cytosol to the plus end via Bik1/CLIP170 (Carvalho et al., 2004; Markus et al., 2011). Thus, the plus-end dynein in yeast is most likely in the open conformation and can interact effectively with dynactin and Num1 (Markus et al., 2020; Marzo et al., 2020). In cultured cells and in reconstituted *in vitro* systems with dynamic microtubules, LIS1 also enhances the plus-end targeting of mammalian dynein, although the plus-end dynein localization also requires dynactin (Splinter et al., 2012; Baumbach et al., 2017; Jha et al., 2017). In the filamentous fungi such as *A. nidulans* and *U. maydis*, the plus-end accumulation of dynein requires kinesin-1 and dynactin but not LIS1 (Zhang et al., 2003; Lenz et al., 2006; Egan et al., 2012). Thus, the plus-end dynein in filamentous fungi could be in the autoinhibited phi conformation before it interacts with LIS1 (Figure 3). In *A. nidulans*, dynein localized along microtubules in cells lacking kinesin-1 is still able to be activated by LIS1 and ΔC-HookA (Qiu et al., 2019), suggesting that LIS1 can also bind to dynein not at the plus end. It cannot be excluded that some LIS1 molecules may bind to dynein during plus-end-directed transport mediated by kinesin-1, and in that case, dynein at the plus end is in the open conformation, waiting to be activated by the early endosome cargo.

When bound to dynactin and cargo adapters, dynein carrying LIS1 at its motor ring is able to undergo processive movement toward the microtubule minus end (Baumbach et al., 2017; Gutierrez et al., 2017), which differs from the inhibitory function of LIS1 on dynein alone (Yamada et al., 2008; McKenney et al., 2010; Huang et al., 2012). However, LIS1 tends to dissociate from the motile dynein-dynactin-cargo adapter complex both *in vivo* and *in vitro* (Lenz et al., 2006; Egan et al., 2012; Lammers and Markus, 2015; Jha et al., 2017; Elshenawy et al., 2020; Htet et al., 2020). In the budding yeast, LIS1 has never been observed to co-localize with dynein-dynactin at the cell cortex (Markus et al., 2011). In filamentous fungi such as *U. maydis* and *A. nidulans*, while dynactin remains associated with a motile early endosome after its dynein-mediated movement has been initiated, LIS1 tends to fall off from it (Lenz et al., 2006; Egan et al., 2012). By observing many *A. nidulans* hyphal tip cells of a *nudF*/lis1 deletion mutant in which early endosome motility is rarely observed, it was found that loss of LIS1 does not affect the speed of occasional dynein-mediated transporting events, and thus, LIS1 was considered as an initiation factor (Egan et al., 2012). The idea that LIS1 is only important for transport initiation is consistent with the current results on LIS1 promoting the open dynein state (Qiu et al., 2019; Elshenawy et al., 2020; Htet et al., 2020; Marzo et al., 2020), thereby enhancing the formation of the dynein-dynactin-cargo-adapter complex (Zhang K. et al., 2017). However, the dynein-dynactin-ΔC-HookA complex still forms without LIS1 in *A. nidulans*, suggesting that dynein-dynactin at the microtubule plus end are capable of binding the HookA-linked early endosome but cannot initiate minus-end-directed movement (Qiu et al., 2019). Further studies will be needed to determine the structure of the dynein-dynactin-ΔC-HookA complex isolated from cells with or without NudF/LIS1, as it seems intriguing why such a complex is not capable of leaving the microtubule plus end *in vivo* in the absence of NudF/LIS1. We should also point out that in neurons, while the microtubule-binding domain of the p150 dynactin is only required for transporting initiation from the distal axon containing dynamic MT plus ends (Lloyd et al., 2012; Moughamian and Holzbaur, 2012), LIS1 is additionally required for continued transport in the mid-axon with much more stable MT (Moughamian et al., 2013). Why is LIS1 so critical *in vivo* while the dynein-dynactin-cargo adapter complex can move without LIS1 *in vitro*? We can envision two possibilities. First, the assembly of the dynein-dynactin-cargo adapter complex containing a second dynein, which is promoted by LIS1 (Elshenawy et al., 2020; Htet et al., 2020), leads to a higher force production (Urnavicius et al., 2018; Elshenawy et al., 2020), thereby facilitating the movement of dynein cargoes in a viscous cytoplasm. Second, there could be a negative regulator that keeps dynein at the phi state *in vivo*, which makes LIS1 absolutely necessary to work against such an inhibition. Future studies will be needed to address these possibilities.

## Other Proteins Important for Dynein Activation *in vivo*-Nude and P150 of Dynactin

LIS1’s binding partner NudE and its homologs are also involved in dynein function (Minke et al., 1999; Efimov and Morris, 2000; Feng et al., 2000; Niethammer et al., 2000; Sasaki et al., 2000; Yan et al., 2003; Liang et al., 2004, 2007; Shu et al., 2004; Li et al., 2005; Guo et al., 2006; Stehman et al., 2007; Ma et al., 2009; Lam et al., 2010; Pandey and Smith, 2011; Wang and Zheng, 2011; Zylkiewicz et al., 2011; Wang et al., 2013; Klinman and Holzbaur, 2015; Reddy et al., 2016; Simões et al., 2018). NudE (homologous to Ro11 in *Neurospora crassa*) (Minke et al., 1999) and its interaction with NudF/LIS1 were first identified in *A. nidulans* and in higher eukaryotic model systems (Efimov and Morris, 2000; Feng et al., 2000; Niethammer et al., 2000; Sasaki et al., 2000). In both *A. nidulans* and budding yeast, loss of NudE causes defects in nuclear migration/spindle orientation, but the defects are much milder compared to that caused by loss of LIS1/NudF/Pac1 (Efimov and Morris, 2000; Li et al., 2005). In *A. nidulans*, mammalian cells, budding yeast and Xenopus egg extract, NudE becomes dispensable if LIS1 concentration is increased (Efimov, 2003; Shu et al., 2004; Li et al., 2005; Wang and Zheng, 2011), consistent with a role of NudE in recruiting LIS1 to dynein (McKenney et al., 2010). In *A. nidulans*, NudE is required for ΔC-HookA-mediated dynein activation, and the requirement of NudE for dynein-mediated early endosome transport can be partially bypassed by constitutively opening dynein using the phi mutations (Qiu et al., 2019). Thus, NudE supports LIS1’s function in dynein activation. NudE not only binds LIS1 but also binds dynein (Sasaki et al., 2000; Liang et al., 2004; McKenney et al., 2011; Wang and Zheng, 2011; Zylkiewicz et al., 2011; Nyarko et al., 2012), but intriguingly, NudE and dynactin p150 compete for binding to the N-terminal site of dynein IC (McKenney et al., 2011; Nyarko et al., 2012). Further studies are needed to reveal how these binding events are regulated to allow dynein activation by LIS1, dynactin and cargo adapters.

EM structural analysis suggests that cargo adapter binding to dynactin may change the conformation of p150 dynactin (Urnavicius et al., 2015). Without cargo adapters, p150 proteins can be seen under EM to exist in either folded or more extended conformations (Urnavicius et al., 2015; Saito et al., 2020). In the folded state, p150’s CC1A and CC1B domains contact the pointed-end complex of the Arp1 mini-filament (Urnavicius et al., 2015), and its microtubule-binding domain (MTBD) is most likely folded inside rather than being exposed (Figure 1B). Although only a minority of dynactin complexes contain folded p150 under EM (Urnavicius et al., 2015), the idea that p150 is mainly in a folded state in the absence of cargo adapters is consistent with the observation that isolated dynactin does not bind microtubules *in vitro* in the presence of dynein without cargo adapters (McKenney et al., 2014). Interestingly, the pointed end proteins including p25 interact with both the cargo adapter and p150’s CC1A and CC1B domains (Urnavicius et al., 2015; Qiu et al., 2018), and the binding sites of the dynein cargo adapters BicD2, Hook3, and BicDR1 at the pointed end overlap with the CC1A- and CC1B-binding sites (Urnavicius et al., 2015; Lau et al., 2020). Thus, it seems possible that BicD2, Hook3, BicDR1 or other similar cargo adapters may compete with p150’s CC1A and CC1B domains for binding to the pointed end, thereby forcing p150 to open up (Cianfrocco et al., 2015; Lau et al., 2020). The pointed end protein p25 is likely to be critically involved in this process as it plays a dual role in cargo adapter binding and the regulation of dynactin-microtubule interaction (Qiu et al., 2018). The most recent structural analysis shows that the whole pointed end complex including p25 acts as an interaction hub for cargo adapters and p150 (Lau et al., 2020). Future studies are needed to address whether cargo adapter binding indeed causes p150 to change its conformation, and if so, how this contributes to the process of dynein activation.

## Future Directions

While the dynein field has made significant progress toward understanding how dynein is activated, some basic questions still remain to be answered. For example, what is the physiological significance of cargo adapter-mediated dynein activation? One obvious purpose of such a regulatory strategy could be to allow inactive dynein to be delivered to the microtubule plus end and stay there to receive its cargo rather than leaving prematurely toward the minus end without the cargo. However, could this regulatory strategy also help save the cellular energy currency ATP and ease the cellular burden of using metabolic pathways to generate ATP, especially when food source is limited? In *A. nidulans*, overexpressing ΔC-HookA in phi mutant cells where dynein is constitutively open produces colonies that are nearly inviable (much sicker than the dynein-null mutant) (Qiu et al., 2019). Could this be related to abnormal ATP consumption, which affects other ATP-utilizing processes or causes the overproduction of unhealthy metabolic products (like lactic acid in a skeletal muscle cell)? Currently, the published dynein ATPase activity (∼200 nmol/min/mg dynein) (Mesngon et al., 2006; McKenney et al., 2010) is likely from inactive dynein, and given the cellular ATP concentration of ∼2 nmol/μl, a fungal tip cell with the volume of ∼1 × 10^–7^ μl would need about half a million dynein molecules to consume the total cellular ATP within a minute (if ATP is not generated by metabolic pathways). It does seem unlikely to have so many dynein molecules in a fungal cell to significantly affect the cellular ATP pool. However, if a fully active dynein has a much higher ATPase activity, this number of dynein molecules will become smaller and more reasonable. It is known that the ATPase activity of an activated myosin II motor can be >100× higher than that of an inactive one (Trybus, 1989; Heissler and Sellers, 2016). In this context, it would be worthwhile to measure the ATPase activities of the inactive phi dynein, open dynein and fully activated dynein (with dynactin and cargo adapters).

There are important open questions on the spatial regulation of dynein in live cells. For example, what are the dynein-dynactin conformational states during kinesin-mediated transport to the microtubule plus end and/or at the plus end? While it will be technically challenging to apply structural analysis on live cells to reveal these states, it should be possible to use dynein-dynactin isolated from specific mutants for biochemical, single molecule and structural analyses. For example, in an *A. nidulans* Δ*hookA* mutant without any early endosomal dynein adapters, many dynein and dynactin molecules accumulate at the microtubule plus end, and it will be interesting to determine their conformational states. Similarly, it would be interesting to determine the conformation of dynein-dynactin or dynein-dynactin-cargo-adapter isolated from cells with or without LIS1. Another interesting issue is how kinesin-3 delivers an early endosome to dynein *in vivo* without being a competitor of dynein, given that *U. maydis* Hok1 affects the kinesin-3-early-endosome interaction and the mammalian Hook3 binds both dynein and kinesin-3 (Bielska et al., 2014b; Kendrick et al., 2019; Siddiqui et al., 2019)? In addition, how do the interactions between the microtubule-binding domain of p150 dynactin and differently modified tubulins affect dynein-mediated transport *in vivo* (Barisic and Maiato, 2016; McKenney et al., 2016; Nirschl et al., 2016; Roll-Mecak, 2020)?

One specific question that deserves to be discussed in more detail is how p150 of dynactin is involved in the dynein activation process. The microtubule-binding domain (MTBD) of p150 is needed for the plus-end accumulation of dynactin-dynein (Vaughan et al., 2002; Kim et al., 2007; Yao et al., 2012) and the initiation of minus-end-directed transport, especially in neurons (Lloyd et al., 2012; Moughamian and Holzbaur, 2012), possibly by helping dynein landing on tyrosinated microtubules (McKenney et al., 2016; Nirschl et al., 2016). However, data from multiple labs also suggest that p150 is an allosteric activator of dynein rather than simply a microtubule-tethering factor (Kim et al., 2007; Dixit et al., 2008; Kardon et al., 2009; Tripathy et al., 2014; Feng et al., 2020). The possibility that cargo binding may change p150 conformation is of great interest in this context. Before cargo binding, p150 could be in folded and more extended conformations and these states are in equilibria (Urnavicius et al., 2015; Saito et al., 2020). We speculate that in fungal cells, before the formation of the dynein-dynactin-kinesin-1 complex, the extended p150 allows its MTBD to be exposed to bind microtubule (Yao et al., 2012). This will help recruit dynein to the microtubule, possibly via the p150 (CC1B)-IC interaction (Karki and Holzbaur, 1995; Vaughan and Vallee, 1995; King et al., 2003; McKenney et al., 2011). We speculate that the subsequent binding to kinesin-1 changes this interaction mode and promotes a folded state of p150 to prevent its MTBD from interfering with kinesin-1-mediated transport, although it is unclear how dynein binds dynactin with a folded p150 (Figure 3). We also speculate that after kinesin-1 is dissociated from dynein-dynactin at the plus end, a transient NudE-IC interaction (Efimov, 2003; McKenney et al., 2011; Wang and Zheng, 2011) may prevent p150’s CC1B from binding to IC (McKenney et al., 2011), thereby stabilizing the folded state of p150. Does cargo adapter binding promote the open state of p150 at the microtubule plus end (Figure 3) and allow its CC1B domain to bind dynein IC again (Urnavicius et al., 2015)? If so, how does the p150-IC interaction in the presence of the cargo adapter change the configuration of dynein HC tails to position them along the Arp1 filament, which eventually leads to dynein activation (Zhang K. et al., 2017)?

Related to the questions on the conformational states of dynactin p150, another important question is how dynein stays at the microtubule plus end. Since the binding to a cargo adapter is a prerequisite for dynein activation, dynein is expected to remain at the plus end before cargo binding. However, we do not know exactly how dynein interacts with the microtubule plus end. In the budding yeast, the microtubule-binding domain of dynein HC is not needed for dynein’s plus-end accumulation (Lammers and Markus, 2015), and thus, yeast dynein is most likely retained at the plus end via LIS1 that binds the plus end-tracking protein Bik1/CLIP170 (Lin et al., 2001; Sheeman et al., 2003; Markus et al., 2011). However, the plus-end accumulation of dynein in filamentous fungi does not need LIS1 or CLIP170 homologs (Zhang et al., 2003; Efimov et al., 2006; Lenz et al., 2006; Egan et al., 2012), but it needs the MTBD of p150 (Yao et al., 2012). Nevertheless, if p150 is folded at the plus end before cargo binding, its MTBD is unlikely to be exposed and used for anchoring dynein at the plus end. Thus, we hypothesize that in filamentous fungi, an open dynein (with LIS1 bound) contacts the microtubule plus end directly using its own microtubule-binding domains (Figure 3), and this open dynein is primed for cargo binding and the subsequent minus-end-directed movement. More data will be needed to either support or refute this hypothesis.

Finally, to gain a full picture of dynein regulation *in vivo*, it will also be important to study the new regulators of dynein. A good example for illustrating this point is the recent progress toward understanding LIS1’s mechanism of action, which was possible only after specific cargo adapters were identified and shown to activate dynein. In this context, we should also point out the need of further dissecting how NudE participates in dynein activation (Qiu et al., 2019), as NudE competes with the CC1B domain of p150 for binding to dynein IC (McKenney et al., 2011; Jie et al., 2017). Moreover, is there any negative regulator that helps keep dynein in the autoinhibited conformation *in vivo*? While we still do not know if such a regulator exists for cytoplasmic dynein, a recent study using Tetrahymena has identified a novel axonemal dynein-binding protein, Shulin, as a regulator that keeps axonemal dynein in an inactivate conformation before it is delivered to cilia (Mali et al., 2020). While both biochemical and genetic approaches can be powerful, genetic screens in *A. nidulans* have been highly valuable in identifying new proteins involved in dynein-mediated intracellular transport (Osmani et al., 1990; Xiang et al., 1995; Efimov and Morris, 2000; Yao et al., 2014; Zhang et al., 2014; Salogiannis et al., 2016). Recently, two new proteins, VezA/vezatin and Prp40A/PRPF40A, have been identified in *A. nidulans* as important factors for dynein-mediated early endosome transport (Yao et al., 2015; Qiu et al., 2020). Vezatin was initially identified as a protein involved in stabilizing cell-cell adhesions (Kussel-Andermann et al., 2000), and PRPF40A is homologous to the yeast RNA-splicing factor Prp40 (Kao and Siliciano, 1996). Interestingly, both vezatin and PRPF40A were identified as Arp1-binding proteins in a biochemical pulldown assay (Hein et al., 2015; Qiu et al., 2020). For vezatin, the interaction with Arp1 could be direct as Arp1 (ACTR1A) was identified as a protein in close proximity to vezatin (VEZT) in human cells (Go et al., 2019)^1^. VezA/vezatin in *A. nidulans* is clearly not a cargo adapter like HookA, and intriguingly, it localizes at the hyphal tip in an actin cytoskeleton-dependent fashion (Yao et al., 2015), and how it affects the microtubule plus end-localized dynein-dynactin will need to be addressed. Recently, a forward genetic screen in Drosophila has also identified a vezatin homolog as being important for dynein-mediated axonal transport, and furthermore, a zebrafish vezatin homolog is also involved in a similar dynein-mediated process (Spinner et al., 2020). The mechanisms of actions of these proteins will need to be further studied in different experimental systems.

## Author Contributions

XX and RQ: writing, editing, and making figures. Both authors contributed to the article and approved the submitted version.

## Conflict of Interest

The authors declare that the research was conducted in the absence of any commercial or financial relationships that could be construed as a potential conflict of interest.

## Footnotes

1 cell-map.org
